# Effect of topical application of melatonin on serum levels of C-reactive protein (CRP), interleukin-6 (IL-6) and tumor necrosis factor-alpha (TNF-α) in patients with type 1 or type 2 diabetes and periodontal disease

**DOI:** 10.4317/jced.52604

**Published:** 2015-12-01

**Authors:** Antonio Cutando, Javier Montero, Rafael Gómez-de Diego, María-José Ferrera, Antonio Lopez-Valverde

**Affiliations:** 1 Department of Special Care in Dentistry, School of Dentistry, University of Granada, Granada, Spain; 2 Department of Surgery, School of Dentistry, Faculty of Medicine, University of Salamanca, Salamanca, Spain; 3 Department of Odontology, Faculty of Health Sciences, University of Alfonso X, Villanueva de la Cañada, Madrid, Spain; 4 Pinos Puente Healthcare Centre, Granada-Metropolitan Health District. Granada, Spain

## Abstract

**Background:**

The present clinical trial study was designed to assess the effect of topical application of melatonin on serum levels of tumor necrosis factor-alpha (TNF-α), interleukin-6 (IL-6) and C-reactive protein (CRP) in patients with diabetes and periodontal disease in comparison with healthy controls.

**Material and Methods:**

Serum levels of TNF-α and IL-6 were measured by enzyme-linked immunosorbent assay and CRP by nephelometry by using the proper commercial kits in 30 patients with diabetes and periodontal disease, and also in a control group of 30 healthy subjects. Periodontograms were performed using the Florida Probe®. Patients with diabetes were treated with a topical application of melatonin (1% orabase cream formula) once daily for 20 days. Healthy subjects were treated with a placebo orabase cream.

**Results:**

Patients with diabetes and periodontal disease had significantly higher mean levels of serum TNF-α, IL-6 and CRP than healthy subjects (*P* < 0.001). Following topical melatonin application, there was a statistically significant decrease in the gingival index and pocket depth (*P* < 0.001) as well as a significant decrease in IL-6 and CRP serum levels (*P* < 0.001). Local melatonin application in patients with diabetes and periodontal disease resulted in a significant decrease in CRP and IL-6 serum levels as well as an improvement in the gingival index and pocket depth. Patients with periodontal disease had significantly higher serum CRP, IL-6 and TNF-α values by comparison with healthy subjects.

**Conclusions:**

We conclude that melatonin can modulate the inflammatory action of these molecules in periodontal patients.

** Key words:**Melatonin, periodontal disease, diabetes mellitus, interleukin-6, tumor necrosis factor-alpha, C-reactive protein, inflammatory markers.

## Introduction

Periodontal disease is an inflammatory oral process affecting the alveolar bone, gums, and periodontal ligament (1). Disease status ranges from gingivitis to advanced periodontitis with destruction of connective tissue attachment and alveolar bone which can eventually lead to tooth loss (2). Periodontitis has also been associated with chronic inflammatory conditions including atherosclerosis, cardiovascular disease, rheumatoid arthritis and diabetes mellitus (3-7). Although the precise relationship between systemic conditions and periodontal disease is still unclear, clinical evidence suggests that periodontitis is associated with a systemic host response and a low-level inflammatory state, as assessed by raised serum levels of systemic inflammatory markers (8,9). Severe periodontitis, both in patients with and without diabetes, has been associated with increased serum levels of proinflammatory cytokines and proinflammatory mediators, including several interleukins (IL), such as IL-7, IL-6, IL-1β, tumor necrosis factor-alpha (TNF-α), and C-reactive protein (CRP) (4,10-15). Consequently, it has been suggested that periodontal disease may contribute to general inflammation and the development of inflammatory systemic diseases (4,10-14). Furthermore, patients with rheumatoid arthritis receiving anti-TNF-α medication have better periodontal indices and lower levels of TNF-α within the gingival crevicular fluids, which indicates that suppression of proinflammatory cytokines might prove beneficial in suppressing periodontal disease (15).

It is known that diabetes is a proinflammatory metabolic disorder that increases the production of cytokines such as IL-6, TNF-α and IL-10 (11-14). Furthermore, it has been suggested that a systemic inflammatory state may be involved in the etiology of this illness (16,17). A relationship appears to exist between periodontal disease and several systemic pathologies-including diabetes mellitus-and it could be hypothesized that controlling one of these two pathologies may be beneficial in controlling the other (17). However, we should remember that periodontal disease is an infectious process with an inflammatory reaction that affects diabetes and glycaemia control. Recent studies have demonstrated that periodontitis itself can cause alterations that the presence of diabetes exacerbates (17). Topical application of melatonin improves these biochemical parameters leading us to consider melatonin is useful in treating this pathology (18). The immunomodulatory effects of melatonin have already been established in patients with and without periodontal disease. A recent systematic review has stated that melatonin may suppress the inflammation of the gingiva and periodontum (19).

Melatonin is an indoleamine secreted by the pineal gland following a circadian pattern.

As it is highly lipophylic, melatonin reaches every cell in the organism, and also melatonin concentrations in saliva are between 24% and 33% of those found in plasma joined to plasma proteins (20). A significant positive correlation between salivary and plasma melatonin exists and, by measuring salivary melatonin, oral pathologies can be studied in relation to plasma and salivary melatonin behavior.

The protective role of the melatonin on the periodontal disease has been reported elsewhere (18,20,21). Furthermore, in a clinical study comparing patients with generalized aggressive periodontitis and periodontally healthy subjects, nonsurgical periodontal therapy for ± 14 days was associated with a significant decrease in TNF-α and IL-17 serum levels in the group with periodontal disease [10]. To our knowledge, no study to date has evaluated the potential benefits of melatonin on TNF-α, IL-6 and CRP serum levels in subjects with diabetes and periodontitis. Hence, a cross-sectional study was conducted to determine the effect of topical melatonin application on serum TNF-α, IL-6 and CRP concentrations in patients with diabetes and periodontal disease and in a control group of healthy subjects.

## Material and Methods

-Participants

The study was conducted at the Healthcare Centre in Pinos Puente (Granada, Spain). A total of 30 healthy individuals of both sexes (20 men, 18 women), aged 31 to 68 years (mean ± standard deviation, 47.0 ± 10.3 yrs) without periodontal disease and 30 patients with diabetes and periodontal disease of both sexes (14 men, 16 women) aged 24 to 58 years (mean 43.1 ± 12.4 yrs) were included in the study. There were 17 patients with type 1 diabetes and 13 with type 2 diabetes ( Table 1).

**Table 1:**
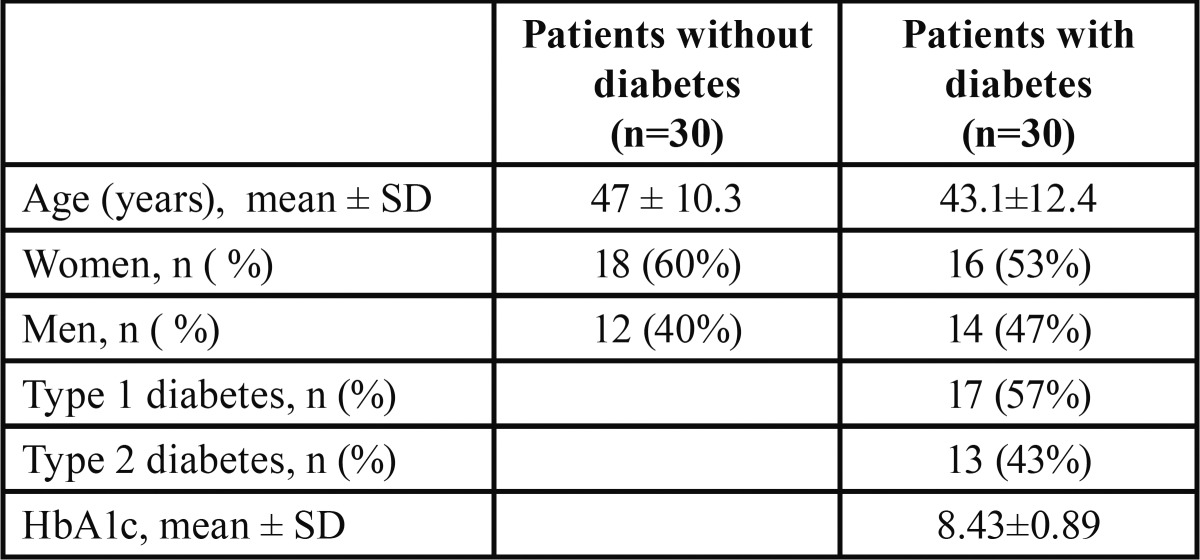
Description of the sample (n=60).

All patients were free of medication (other than diabetes regimens). Type of periodontal disease was not an inclusion criterion, although most patients presented advanced periodontitis (83.3%) and only 5 patients had mild or moderate periodontal disease. Exclusion criteria included current use of bisphosphonates, oral contraceptives, antibiotic treatment in the previous 6 months, and having received (within the last 6 months) or currently being treated for diseases of the oral cavity. All healthy subjects were in good general health with no history of systemic disease or clinical signs of periodontal disease. The study was approved by the Ethics Committee of the Faculty of Dentistry of the University of Granada (Spain). Written informed consent was obtained from all participants.

-Study Procedures

All participants (patients and controls) underwent an oral examination, including medical, dental, and caries assessments. The same dentist performed all examinations. Periodontograms were performed using the Florida Probe® handpiece (computerized periodontal probing system). The gingival index and probing depth were recorded in patients with diabetes and in healthy subjects. The diagnosis for periodontal disease was based on probing periodontal pockets ≥ 4mm.

The gingival inflammation was evaluated by probing of the gingival margin and observing the spontaneous bleeding of the gingiva.

Thereafter, patients with diabetes were treated with a topical melatonin application (1% orabase cream formula) in the upper and lower dental arches on attached gingival surfaces for 20 days. Pure melatonin was purchased from Helsinn Advanced Synthesis, SA (Biasca, Switzerland). Melatonin 1% orabase cream formula was prepared by the Department of Hospital Pharmacy of the Hospital Perpetuo Socorro (Granada, Spain). Quality was certified by Methapharmaceutical Ind. (Barcelona, Spain). Orabase composition: Sodium Carboximetilcelulosa16.5%; Pectin 16.5%; Jello 16.5%; Plastibase c.s.p.100.

Each patient was given the same kind of toothbrush, with the same surface, explaining each patient had to place the pasta with melatonin, without exceeding the limits of the toothbrush. The bore diameter of the tube outlet cream was exactly the same in all samples. The exact dosage, is achieved by means of a simple dispenser, applied to the tube outlet, which supplies a 1cc amount of melatonin vs. placebo preparation.

Healthy subjects were treated with a placebo orabase cream. Melatonin cream (or the placebo) was applied daily at night after routine oral hygiene. All participants were instructed how to use the orabase cream. It was recommended they apply the amount that fits on a regular-size adult toothbrush to each dental arch. Furthermore, all participants received the same brand of toothpaste for use during the course of the study. Conventional periodontal treatment prior to or during the study was not allowed. Oral examinations were also performed at the end of treatment (20 days after).

-Measurements of TNF-α, IL-6 and CRP Serum Concentrations

In all participants (patients with diabetes and periodontal disease and healthy controls), blood samples were collected in fasting conditions at baseline and after treatment with melatonin or the placebo. Serum was separated from blood by centrifuging (10 min at 1300 rpm), stored in aliquots at -80°C, and thawed immediately before assay. Aliquots of each sample were assayed using commercially available TNF-α and IL-6 sensitive ELISA (R & D Systems Inc., Minneapolis, MN, USA) in accordance with the manufacturer’s instructions. The lower limits of detection were 0.016 pg/mL for IL-6 and 0.06 pg/mL for TNF-α. The CRP concentration in serum was measured using nephelometry, a commercially available latex particle-enhanced immunoassay (NA Latex CRP kit, Dade Behring, Tokyo, Japan).

-Plasma melatonin determination

Patients came to the laboratory at 08:00 after an overnight fast and remained seated for 30 min before samples were taken. Blood samples were drawn from the antecubital vein and centrifuged at 3000 g for 10 min. Plasma was separated and frozen at -20ºC until analysis. Plasma melatonin was determined by a commercial RIA (DVD Biochemie, Marburg GmbH, Germany) and quality control was performed showing intra- and inter-assay coefficients of variation of 11.3% and 6.3%, respectively. The recovery of added melatonin was 84.4% and assay sensitivity was 4.65 pg/mL.

-Statistical Analysis

Quantitative variables are expressed as mean ± standard deviation (SD). The paired Student’s t test was used to compare the gingival index and probing depth before and after topical application of melatonin in patients with diabetes and periodontal diabetic disease, and the Student’s t test for independent samples to compare TNF-α, IL-6 and CRP serum levels between patients with diabetes and healthy subjects. The relationship between the gingival index and probing depth with TNF-α, IL-6 and CRP serum levels was assessed with Pearson’s correlation coefficient. Statistical significance was set at *P* < 0.05. SPSS 11.0 was used to analyze data.

## Results

Comparison of TNF-α, IL-6 and CRP serum levels in patients with diabetes + periodontal disease and healthy controls before topical treatment with melatonin is shown in  Table 2. Patients with diabetes and periodontal disease had significantly higher mean levels of serum TNF-α (1.79 ± 0.19 vs 0.82 ± 0.17 pg/mL), IL-6 (0.57 ± 0.07 vs 0.38 ± 0.05 pg/mL), and CRP (0.39 ± 0.11 vs 0.21 ± 0.08 mg/L) by comparison with healthy subjects. Moreover patients with diabetes had significantly lower values of salivary melatonin (4.5 ± 0.81 vs 2.7 ± 0.81 pg/mL) and plasma melatonin (13.9 ± 3.87 vs 9.7 ± 3.27 pg/mL) than healthy subjects.

**Table 2:**
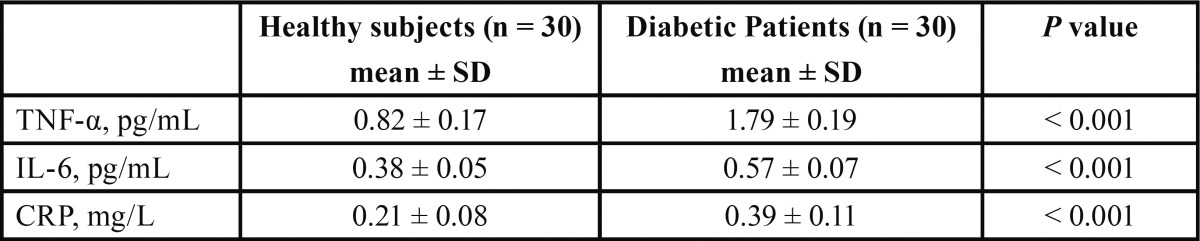
Comparison of serum TNF-α, IL-6 and CRP levels in diabetic patients with periodontal disease and healthy controls before topical treatment with melatonin.

In periodontal terms, after a daily topical melatonin application during 20 days among diabetic+periodontal patients, there was a statistically significant decrease in the gingival index (15.84 ± 10.26 vs 5.59 ± 4.08) and pocket depth (28.29 ± 19.48 vs 11.90 ± 9.01). Moreover it was observed a significant decrease in serum levels of IL-6 (0.57 ± 0.07 vs 0.47 ± 0.07 pg/mL) and CRP (0.39 ± 0.11 vs 0.31 ± 0.11 mg/L). However, no significant differences were found in serum TNF-α levels before and after treatment with topical melatonin ( Table 3).

**Table 3:**
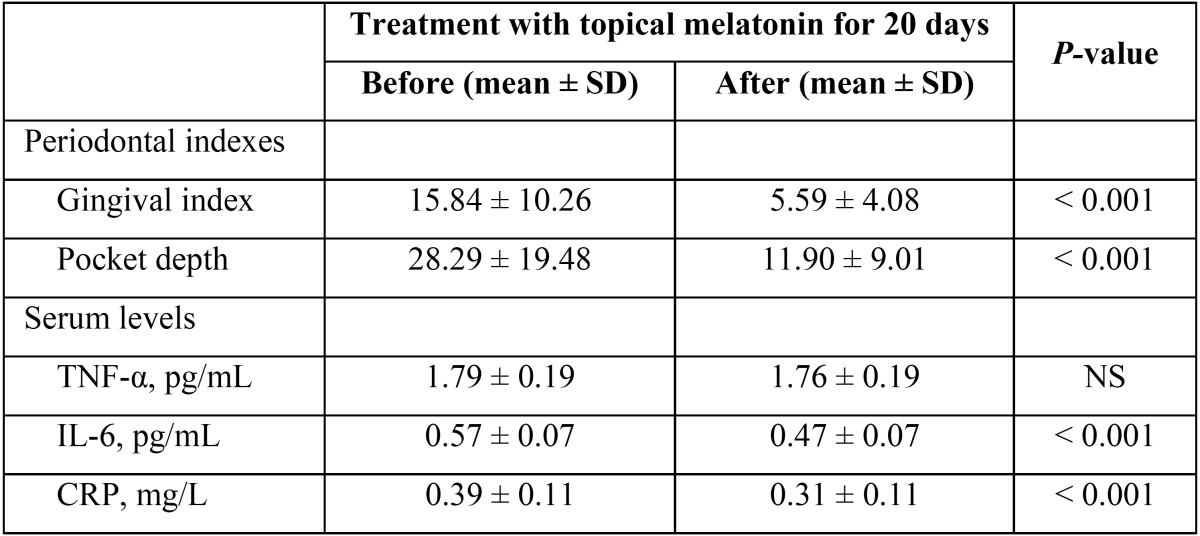
Comparison of gingival index, pocket depth and TNF-α, IL-6 and CRP serum levels before and after topical application of melatonin in patients with diabetes and periodontal disease.

Furthermore, it was found that TNF-α, IL-6 and CRP of serum levels before and after topical melatonin treatment were significantly correlated with the gingival index and pocket depth before and after treatment ( Table 4). This means that a decrease in the gingival index and pocket depth was linearly proportional with the decrease in TNF-α, IL-6 and CRP serum levels after topical melatonin treatment.

**Table 4:**
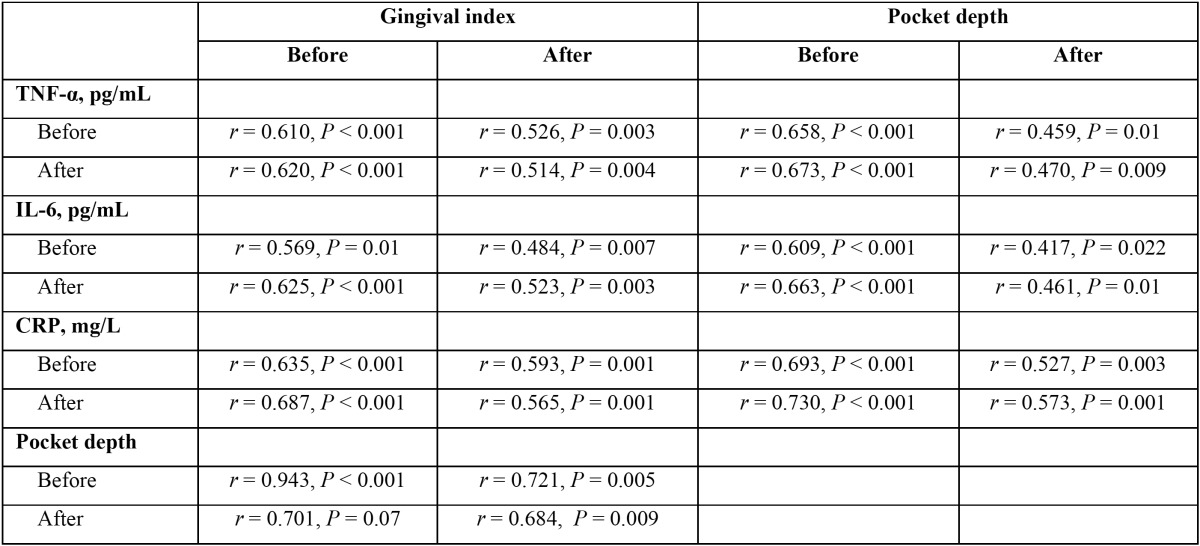
Correlations between gingival index, pocket depth and TNF-α, IL-6 and CRP levels in serum before and after treatment with melatonin in the patient group (n=30).

## Discussion

The present results confirm the beneficial effect of topical melatonin application on clinical parameters of periodontal disease, such as the gingival index and pocket depth. Moreover, it was confirmed that the severity of the periodontal disease and the serum levels TNF-α, IL-6 and CRP were significantly correlated. Before melatonin treatment, serum TNF-α, IL-6 and CRP levels in diabetic patients with periodontal disease were significantly higher than in healthy control subjects. It should be taken into account that human adipose tissue is a potent source of inflammatory interleukins that could cause a false relationship between periodontal illness and the presence of interleukins (22). After topical melatonin treatment, there was a statistically significant decrease in the gingival index and pocket depth, as well as a significant decrease in serum leves of IL-6 and CRP. This, together with the fact that the melatonin was used in topical form, supports our belief that the origin of the interleukins is, basically, a consequence of periodontal disease. The results obtained by using topical melatonin appear to be better than those obtained by other authors using nonsurgical treatment of periodontal disease (23). However, not all authors agree that treating periodontal disease reduces interleukin concentrations in serum (24).

The present results demonstrate that diabetic patients with untreated periodontitis present increased circulating levels of markers of inflammation and proinflammatory cytokines (CRP, TNF-α and IL-6). Over expression of these inflammatory mediators in serum in subjects with periodontal disease suggests a potential hyper-reactivity of cells in these individuals that may favour periodontal tissue breakdown and a systemic inflammatory burden. Elevated levels of cell-mediated and cytokine-mediated markers of inflammation-including CRP and other cytokines-are reported to be associated with periodontal disease (10). In systematic reviews and meta-analyses of CRP protein in relation to periodontitis, CRP levels were consistently higher in patients than in controls (25,26). It has been estimated that the weighted mean difference in CRP between patients and controls was 1.56 mg/L (*p* < 0.00001) and that the levels of CRP after periodontal therapy, is reduced on average 0.50 mg/L (95% confidence interval [CI] 0.08-0.93) (25). Moreover, a meta-analysis of 4 randomized clinical trials (26) estimated a significant reduction in CRP levels (-0.231 mg/L on average) after introducing periodontal treatment, which indicates that nonsurgical periodontal treatment had a positive effect on reduction of serum CRP levels. The present results agree with findings from both meta-analyses, although in our study periodontal treatment was replaced by topical application of melatonin, the beneficial effect of which on periodontal disease has been demonstrated in previous studies (20,21,27).

The systemic levels of TNF-α in subjects with periodontitis and the impact of periodontal treatment on serum TNF-α concentrations are little understood. We found significantly higher TNF-α levels among patients with diabetes and periodontal disease than in healthy subjects however, the TNF-α levels before and after melatonin use did not change significantly, therefore this serum parameter seems to be indifferent to the periodontal changes, in contrast to that reported elsewhere (28). Higher levels of circulating TNF-α in patients with periodontitis as compared with healthy subjects have consistently been reported in most studies (10,24). However, the effect of periodontal therapy on the systemic level of TNF-α has not been clarified. Mendes Duarte *et al.* (10) report that subjects with generalized aggressive periodontitis presented higher levels of circulating TNF-α than controls but TNF-α levels remained higher after periodontal therapy. Nakajima *et al.* (29) report that periodontal treatment did not affect TNF-α levels 

We are aware that Type I diabetes, is more aggressive and could be a determining factor to the effect of melatoniona, but the sample size is insufficient to find significant differences between these subgroups.

As reported elsewhere we found high levels of IL-6 in patients with periodontal disease when compared with healthy controls. The IL-6 is an important proinflammatory cytokine involved in the regulation of host response to tissue injury and infection. It is produced by a variety of cells, such as monocytes, fibroblasts, osteoblasts and vascular endothelial cells, in response to inflammatory challenges (27).

Subjects included in the study were not instructed to change their habits of toothbrushing, therefore, we believe that the results are attributable to the effect of melatonin, but it is possible that, some subjects, change their oral hygiene habits when they entered the study; in fact, some studies show a reduction of IL-1ß, only with changing brushing techniques (30).

Future studies, should include positive and negative controls and tests, ie healthy patients with periodontitis and diabetic patients without periodontitis, to avoid confusion of diabetes factor in the outcome (periodontitis).

To summarize, the diabetic patients with periodontal disease had significantly higher serum CRP, IL-6 and TNF-α values by comparison with healthy subjects. After a 20 days of daily local melatonin application in such diabetic patients with periodontal disease, a significant decrease in serum CRP and IL-6 levels as well as an improvement in the gingival index and pocket depth was observed.
